# Evaluation of Urinary Catheterization Competency and Self-Efficacy in Nursing Students Using the Flipped Classroom Approach

**DOI:** 10.3390/ijerph192215144

**Published:** 2022-11-17

**Authors:** Gabriel Aguilera-Manrique, Lorena Gutiérrez-Puertas, Vanesa Gutiérrez-Puertas, Blanca Ortiz-Rodríguez, Verónica V. Márquez-Hernández

**Affiliations:** 1 Department of Nursing, Physiotherapy and Medicine, Faculty of Health Sciences, University of Almeria, 04120 Almería, Spain; 2 Research Group of Health Sciences, CTS-451, Universidad de Almeria, 04120 Almería, Spain; 3 Health Research Centre, University of Almeria, 04120 Almeria, Spain; 4 Research Group Experimental and Applied Neuropsychology, HUM-061, Universidad de Almeria, 04120 Almería, Spain; 5 Research Group for Electronic Communications and Telemedicine, TIC-019, Universidad de Almeria, 04120 Almeria, Spain

**Keywords:** flipped classroom, nursing students, self-efficacy, skills, urinary catheterization

## Abstract

Urinary catheterization is a complex procedure. Traditional teaching in classroom, such as using a simulator, is not enough to guarantee the necessary learning outcomes. It is therefore necessary to implement new active learning approaches such as the flipped classroom. The aim of this study was to examine the effect of the use of the flipped classroom on the level of self-efficacy and the assessment of clinical competencies in nursing students performing the urinary catheterization procedure. A quasi-experimental study of a single group with pre-/post-testing measures. A convenience sample of 139 nursing students. A flipped classroom approach was implemented. Measurements were taken pre and post intervention. This study followed the consolidated criteria for quality of nonrandomized evaluations studies (TREND) checklist. Regarding self-efficacy, statistically significant differences were found between the pre-test and post-test (Z = −14.453; *p* < 0.05). With respect to knowledge level, statistically significant differences were found when comparing pre-/post-test results (Z = −14,480; *p* < 0.05). Furthermore, statistically significant differences were found on the ability scale depending on pre-/post-test (Z = −10.277; *p* < 0.005); in all cases, obtaining a higher score in the post-test. The flipped classroom could be an effective educational tool for the development of clinical skills, specifically urinary catheterization. This method has been shown to improve the knowledge, skills, and self-efficacy in urinary catheterization of nursing students.

## 1. Introduction

Nursing students within nursing programs face the great challenge of having to master both educational content and clinical skills [1]. Almost half of nursing education is based on practical training [2]. The clinical environment also allows students to put into practice techniques which they have learned in the nursing practice laboratories [3]. It is therefore necessary to have adequate knowledge and good training in clinical skills to ensure patient safety and increase students’ self-confidence when carrying out clinical procedures on patients [4]. This highlights the importance of clinical skills training in the nursing practice laboratory [5].

Urinary catheterization is a particularly complex and invasive procedure which can lead to complications such as trauma to the bladder or urethra through making an incorrect insertion or by introducing microorganisms into the urinary system which can trigger an infection [6]. According to evidence-based guidelines, it is thus necessary to have the knowledge and ability to be able to prevent complications and guarantee patient safety [7]. Conversely, traditional teaching of this procedure has been shown to be efficient, but it does not achieve the necessary learning outcomes [8]. Other learning methods of urinary catheterization have been explored, such as virtual reality, but have not been shown to be more efficient than traditional methods [9]. For this reason, identifying and developing adequate teaching and learning strategies to improve nursing students’ abilities, knowledge and self-efficacy poses a challenge for nurse educators [5].

On the other hand, self-efficacy is an important element in increasing self-confidence in nursing students as they acquire clinical knowledge and skills [10]. Self-efficacy refers to the capacity perceived by individuals to achieve a level of performance in a specific area, bridging the gap between knowledge and action [11]. Several studies have explored the influence of self-efficacy on the academic and clinical performance of nursing students, considering it a precursor to success [12,13]. In particular, the self-efficacy of nursing students is connected to the development of their knowledge and skills for performing their postgraduate duties successfully in a clinical setting [1,14]. In addition, assessing the self-efficacy of clinical skills allows the students to evaluate their performance and learning experience [15].

In terms of preparing nursing students for clinical practice, curriculum and delivery should promote student participation, active learning and the development of self-efficacy [16]. However, traditional education is inadequate for meeting these requirements [17]. It is therefore necessary to implement new active learning methods which involve students in their training with the aim of improving self-efficacy in clinical skills [14]. The flipped classroom (FC) is a new teaching method which provides the opportunity to bridge the gap between education and practice for nursing students [18]. The FC also allows for teamwork and active participation in learning, these being important aspects in providing nursing students the skills and competencies required for working in the clinical environment [19]. In this educational methodology, students prepare the core content through pre-class activities, such as reading a chapter of a book or article or watching a video or presentation. Students then implement the core content in multiple ways in the classroom, such as through clinical cases, problem-solving or clinical skills practice [20].

The FC has been shown to improve academic performance [21], self-directed learning ability, critical thinking and self-efficacy [22]. Likewise, various studies indicate that students view the implementation of this methodology positively [23,24]. Other studies, however, have found that student satisfaction is relatively low [25,26]. On the other hand, the impact that the FC has on nursing students’ clinical skills has not yet been investigated in great detail, so more conclusive evidence is required to determine this [27]. In particular, these studies suggest that the FC is more effective than traditional teaching in improving theoretical knowledge [28], and the acquisition of clinical skills [29]. Nevertheless, studies have not yet been conducted which examine the effect of using the FC for training in clinical skills, such as urinary catheterization.

In summary, nurse educators should implement educational techniques to improve self-efficacy in nursing students’ clinical skills, with the aim of enhancing their performance in the clinical setting. Consequently, the aim of this study was to examine the effect of the use of the flipped classroom on the level of self-efficacy and the assessment of clinical competencies in the urinary catheterization procedure performed by nursing students.

## 2. Materials and Methods

### 2.1. Design and Participant

**Figure 1:**
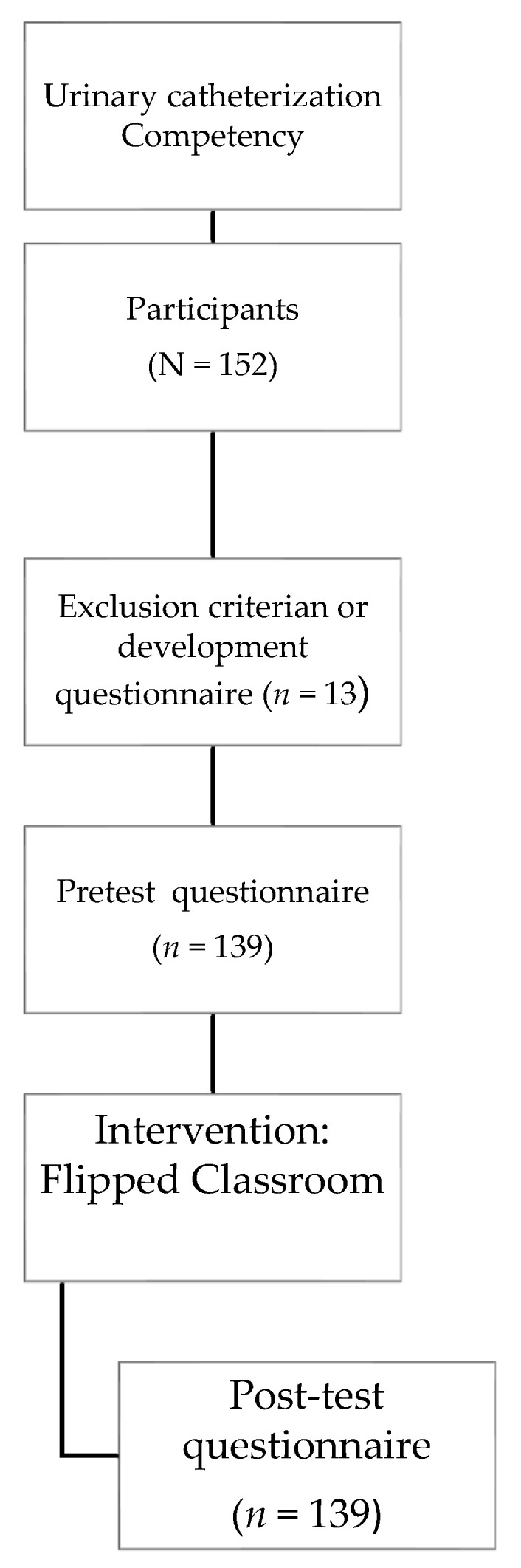
Flow diagram of participants.

The study was quasi-experimental non-randomized, pre-test post-test design, carried out on a single group. A total of 139 students participated in total and were selected through a convenience sample. Being matriculated in the Adult Nursing I course was considered as an inclusion criterion, while being an exchange student was considered to be an exclusion criterion, because they may not have possessed a sufficient level of the native language used in the study, so as not to interfere with the understanding of the intervention and instruments used (Figure 1). This study followed the consolidated criteria for quality of nonrandomized evaluations studies (TREND) checklist.

### 2.2. Instruments

Firstly, socio-demographic data were obtained, such as age, sex, previous studies, previous experience in hospitals or residences, whether participants had seen other professionals perform urinary catheterization, and whether they had had previous opportunities to perform the procedure. An ad hoc self-efficacy scale was developed, consisting of 35 items which the participant had to score from 0–100, depending on their confidence level when performing the steps of the urinary catheterization procedure. The higher the score, the higher the level of self-efficacy. Cronbach’s alpha of the self-efficacy scale was 0.949. A knowledge questionnaire consisting of 10 questions on the bladder catheterization procedure was completed. Each answer was awarded a score of 0 to 1 depending on whether it was incorrect or not, respectively. The higher the score, the higher the level of knowledge. Cronbach´s alpha of the knowledge questionnaire was 0.744. An observation scale was used for the ability to conduct the urinary catheterization procedure. The scale consisted of 27 items for the steps of the procedure, with a dichotomous yes/no response and a score of 1/0, respectively. The results were distributed across the following ranges: 0–9: low skill, 10–18: average skill, and 19–27: high skill. Cronbach’s alpha of the scale was 0.811. The instruments have been developed by a panel of experts made up of nursing professionals and university professors in the nursing field with more than 10 years of experience. For the development of the instrument, previous research related to the acquisition of clinical competencies in nursing students and recommended practices for the performance of bladder catheterization was consulted in order to assess the knowledge, skills and self-efficacy of nursing students in the performance of bladder catheterization. Content validity was assessed by a panel of experts. Scores and cut-off points were established based on previous studies. After the development of the questionnaire, a pilot test was carried out with 10 participants who met the inclusion and exclusion criteria established in the study, who subsequently did not form part of the study participants. After completing the questionnaire, participants were briefly interviewed to assess their understanding and applicability of the questionnaire. The research team members analyzed and discussed the information gathered from the interviews to determine whether to make modifications.

### 2.3. Data Collection

First of all, permission was requested from the University Institutional Review Board, and once this was obtained, students enrolled in the Adult I course were invited to an explanation of the research aim. Students interested in participating signed the informed consent form and then completed questionnaires on knowledge and self-efficacy in performing urinary catheterization. In addition, they were shown the self-study tasks to be completed before the face-to-face session a week in advance. They were provided an online link to the chapter of the book dealing with bladder catheterization and a video that had been recorded by the nurse educator. The students were then organized into 10 groups of around 13 or 14 students to attend the face-to-face session. The students had to prepare a PowerPoint presentation to present in the face-to-face session. In the first face-to-face session, two assessors evaluated the students’ skills by completing a checklist on an individual basis for the urinary catheterization procedure. The PowerPoint presentation followed the evaluations and at the end of the presentation a discussion of the topic was initiated, doubts were addressed and learning experiences were shared. The nurse educator then answered the questions raised during the discussion and presented a summary of the theoretical contents and a classroom demonstration of urinary catheterization. The students’ presentations were uploaded for one week to an online learning forum where they could continue to ask questions, so that all groups could see the presentations developed in the other groups. After one week the students attended another face-to-face session, filling in the knowledge and self-efficacy questionnaires. Two assessors examined the students’ skills in performing urinary catheterization individually using a checklist. When all the students in the group had finished, they met, and a debriefing was conducted. The data collection took place between January to March 2021.

### 2.4. Ethical Considerations

The study was approved of by the University Institutional Review Board (EFM-62/20). The participants were informed of the aim of the study, the anonymous and confidential treatment of data, as well as the possibility to leave the study at any time. The students who wanted to participate had to sign the informed consent form and they were reminded of the voluntary nature of their participation. The guidelines established in the Helsinki declaration were followed at all times.

### 2.5. Data Analysis

The statistical program SPSS version 27 was used for data analysis. For the descriptive analysis, the mean and standard deviation of the quantitative variables were analyzed, while for the categorical variables, frequencies and percentages were obtained. We used the Wilcoxon test to compare pre-/post-test results, the non-parametric Mann–Whitney U test and the Spearman correlation coefficient were used. For the evaluation of intra- and interobserver consistency, Cohen’s Kappa coefficient was calculated. A *p* < 0.05 was considered significant.

## 3. Results

### 3.1. Socio-Demographic Characteristics of the Sample

From the total number of participants (N = 139), 78.4% (*n* = 109) were women and 21.6% (*n* = 30) were men. The mean age of participants was 21.02 ± 6.11. In terms of previous studies, most participants had completed the last two years of high school and 17.3% (*n* = 24) had had previous experience in hospitals or residences.

Regarding urinary catheterization specifically, 18.7% (*n* = 26) had witnessed other professionals carry out a catheterization. Conversely, most participants (97.8%, *n* = 136) had not had the opportunity to carry out the procedure. Table 1 details the socio-demographic characteristics of the sample.

### 3.2. Self-Efficacy Scale

With respect to self-efficacy, statistically significant differences were found between pre-test and post-test (Z = −14.453; *p* < 0.05). Specifically, at the pre-test the participants obtained a mean percentage of 62.59 ± 17.58 points, while in the post-test the mean was 89.36 ± 9.97. In Table 2 the results of the scores for each self-efficacy item can be seen in detail.

Statistically significant differences were not found when comparing the post-test results in terms of sex (U = 1551.500; Z = −0.428; *p* = 0.669) or age (rs = −0.045; *p* = 0.603). However, statistically significant differences were found with regards to having had the opportunity to observe other professionals carry out the procedure (U = 5484.000; Z = −0.75; *p* < 0.05). Participants who had seen other professionals perform a catheterization obtained the highest scores in self-efficacy.

### 3.3. Knowledge Questionnaire

Considering the level of knowledge, statistically significant differences were found when comparing the pre-/post-test results (Z = −14.480; *p* < 0.05). At the time of the pre-test, the participants obtained an average score of 4.29 ± 1.54, while in the post-test they obtained a score of 6.26 ± 1.72. Statistically significant differences were not found at the time of the post-test with regards to the sex of participant (U = 1579.000; Z = −0.291; *p* = 0.771), and neither were statistically significant differences found in terms of age (rs = 0.061; *p* = 0.474).

### 3.4. Ability Scale

Statistically significant differences were found in the ability scale depending on whether it was at the time of the pre-test or post-test (Z = −10.277; *p* < 0.005). At the pre-test, the participants obtained a mean score of 18.25 ± 3.36 (average ability), whereas in the post-test they received a mean score of 22.32 + 1.12 (high ability). The congruency between the observation measures was intraobserver (0.816) and interobserver (0.891).

## 4. Discussion

The aim of this study was to examine the effect of the use of the flipped classroom on the level of self-efficacy and the assessment of clinical competencies in the urinary catheterization process in nursing students. As far as the implementation of the FC is concerned, an improvement in self-efficacy in nursing students was observed. Various studies have shown an increase in self-efficacy following the implementation of the FC [30,31]. Furthermore, the data have shown that the FC increases knowledge and skills in nursing students in performing urinary catheterization. These findings are consistent with those reported in other studies on clinical abilities carried out on nursing students, where a significant increase in knowledge and clinical skills was observed through the use of the FC, considering it more effective than traditional teaching [28,29]. The increase in knowledge, clinical skills and self-efficacy may be due to previously viewing videos of the implementation of urinary catheterization, as shown in previous studies on clinical skills [5,32]. In addition, the videos enable the repeated viewing of the procedure, allowing nursing students to transfer their knowledge of urinary catheterization from their short-term memory to long-term memory [33]. A further aspect that may have had an influence is the flexible learning approach, which allows students to access the resources when and where they want, thus satisfying their educational needs at any time [34]. Additionally, the discussion group favors communication and teamwork among the peers and with the nurse educator, allowing the educator to identify weaknesses and aspects to consider for the improvement of students’ training [35]. On the other hand, concerning self-efficacy, it was observed that nursing students who had observed other professionals, or who had had the opportunity to perform a urinary catheterization in patients, scored more highly in this aspect. These data are consistent with those of other studies [5,36]. In the same vein, by applying this theory, nursing students are able to successfully develop their clinical skills through experience and observation, increasing their self-efficacy in the mastery of clinical skills [3].

Regarding the assessment of clinical skills and self-efficacy in urinary catheterization, it could be the basis for identifying gaps in knowledge, leading to further training. This assessment allows for the improvement of clinical skills before performing the procedures on patients [32]. Furthermore, nursing students with greater competency in psychomotor skills are able to develop the essential critical judgment and cognitive skills required for administering quality and safe care in the clinical setting [37]. Along the same lines, other studies have indicated an improvement in the skills and knowledge score following the implementation of the FC [38,39], consistent with those obtained in this study.

### Limitations

The findings from this study should be considered taking into account a series of limitations. Firstly, the sample was selected through convenience sampling, and in a single institution, which makes it difficult to generalize the findings. In addition, some participants prior to the study had seen the urinary catheterization procedure or had previous experience in a clinical setting, which may have influenced knowledge and self-efficacy through prior vicarious learning. On the other hand, the tools used are self-reported, there is a possibility of social desirability. In this case, the inclusion of skills assessment by external observers decreases this limitation and increases the reliability of the data.

## 5. Conclusions

The FC has been demonstrated to be an effective educational approach to enable the acquisition of clinical competencies, specifically in urinary catheterizations. This method has been shown to improve knowledge, skills, and self-efficacy in nursing students. This intervention can bridge the gap between theory and practice, improving nursing students’ performance in the clinical setting. Urinary catheterization is a complex and invasive procedure that can lead to complications such as infection if the technique is not performed correctly. Therefore, it is important to train students to acquire competencies in urinary catheterization. The FC allows students to be an active element in the training process. The implementation of FC as an educational methodology for the acquisition of knowledge, skills, and self-efficacy for urinary catheterization could be a suitable resource for nursing students. Moreover, it would be interesting to carry out research comparing the flipped classroom with other teaching methods used for the acquisition of clinical competencies of nursing students. In addition, qualitative studies could be developed to learn about students’ perceptions of FC as a methodology for the acquisition and evaluation of competencies.

## Figures and Tables

**Table 1:** Socio-demographic characteristics of the sample.

Variable	Total (N = 139)	
	*n*	%
Sex		
Male	30	21.6
Female	109	78.4
Age	21.02 *	6.11 **
Previous studies		
Completed high school	114	82
Professional training	23	16.5
University degree	2	1.4
Previous experience in hospitals or residences		
Yes	24	17.3
No	115	82.7
Had seen a professional perform a urinary catheterization		
Yes	26	18.7
No	113	81.3
Had previous opportunity to perform a urinary catheterization		
Yes	3	2.2
No	136	97.8

* Mean, ** Standard Deviation.

**Table 2:** Results of the self-efficacy pre-/post-test.

Item	Pre-Test		Post-Test	
	M *	SD **	M *	SD **
1. Identify types of urinary catheterization	32.55	27.42	71.37	25.25
2. Check the patient’s identity	95.32	10.85	97.19	8.68
3. Check for allergies (latex)	76.22	27.032	84.82	23.23
4. Inform the patient of the procedure and clear up any doubts	63.35	32.10	87.59	15.97
5. Maintain privacy	90.04	15.61	96.26	8.62
6. Put the patient in the appropriate position (male or female)	62.52	33.03	95.40	10.91
7. Hygienic hand washing	96.12	9.59	97.77	6.37
8. Put on non-sterile gloves	90.50	13.63	95.14	10.37
9. Prepare the material for genital sanitation	57.27	31.24	90.43	14.63
10. Antiseptic handwashing	88.49	19.14	94.32	12.22
11. Put on sterile gloves (insertion)	79.10	21.06	92.37	12.75
12. Prepare the materials for the catheter insertion	49.86	33.62	90.18	13.49
13. Choose the appropriate catheter, type and caliber	31.58	28.137	70.04	21.89
14. Place the sterile cloth around the genital area	57.84	33.63	93.71	11.48
15. Check the balloon for reliability	46.96	33.19	92.66	14.06
16. Remove the content introduced	38.20	31.30	96.65	87.40
17. Connect the catheter to the drainage system	44.96	33.73	90.51	14.78
18. Perform asepsis of the area	51.83	32.39	88.85	16.15
19. Lubricate the proximal part of the catheter with an adequate amount of lubricant	62.48	30.29	94.24	9.47
20. Insert the catheter appropriately	30.22	28.70	78.88	17.96
21. Check that the catheter balloon is inside the urinary bladder	30.72	29.33	82.30	17.70
22. Introduce the quantity of double-distilled water into the balloon	36.33	32.55	86.19	17.042
23. Check the attachment of the catheter to the urinary bladder	30.65	27.77	84.75	18.74
24. Clean any excess lubricant from the genitals	68.92	27.54	95.68	78.97
25. In non-circumcised male patients, place the foreskin in the correct position	49.35	34.99	85.32	19.31
26. Remove material from around the patient	77.34	28.14	93.45	13.11
27. Secure the catheter to the appropriate place	43.81	33.60	87.23	17.27
28. Fasten the bag to its support and to the side of the bed	57.19	36.09	92.45	12.73
29. Gather up the materials	86.04	19.95	94.39	10.84
30. Make the patient comfortable	81.94	22.83	93.45	10.54
31. Dispose of the materials	86.04	21.08	93.81	14.07
32. Take off gloves and wash hands	95.11	10.17	95.40	13.20
33. Log the procedure	88.67	80.37	89.28	17.51
34. Remove the catheter adequately	39.78	31.74	80.58	19.62
35. Indicate your level of self-confidence in correctly carrying out the procedure	42.29	23.57	75.04	14.39

* Mean, ** Standard Deviation.

## Data Availability

Data available on request due to restrictions.

## References

[1] Van Horn E., Christman J. (2017). Assessment of nursing student confidence using the clinical skills self-efficacy scale. Nurs. Educ. Perspect..

[2] McNett S. (2012). Teaching nursing psychomotor skills in a fundamentals laboratory: A literature review. Nurs. Educ. Perspect..

[3] Ewertsson M., Allvin R., Holmström I.K., Blomberg K. (2015). Walking the bridge: Nursing students’ learning in clinical skill laboratories. Nurse Educ. Pract..

[4] Kol E., Ince S., Işik R.D., Ilaslan E., Mamakli S. (2021). The effect of using standardized patients in the Simulated Hospital Environment on first-year nursing students psychomotor skills learning. Nurse Educ. Today.

[5] Chuang Y.H., Lai F.C., Chang C.C., Wan H.T. (2018). Effects of a skill demonstration video delivered by smartphone on facilitating nursing students’ skill competencies and self-confidence: A randomized controlled trial study. Nurse Educ. Today.

[6] Öztürk D., Dinç L. (2014). Effect of web-based education on nursing students’ urinary catheterization knowledge and skills. Nurse Educ. Today.

[7] Beggs-Yeager C., Sharts-Hopko N., McDermott-Levy R. (2021). The role of nurses in surveillance to enhance global health security: A delphi study. Nurs. Outlook.

[8] Kardong-Edgren S., Mulcock P.M. (2016). Angoff method of setting cut scores for high-stakes testing: Foley catheter checkoff as an exemplar. Nurs. Educ..

[9] Kardong-Edgren S., Breitkreuz K., Werb M., Foreman S., Ellertson A. (2019). Evaluating the usability of a second-generation virtual reality game for refreshing sterile urinary catheterization skills. Nurs. Educ..

[10] Stump G.S., Husman J., Brem S.K. (2012). The nursing student self-efficacy scale: Development using item response theory. Nurs. Res..

[11] Bandura A. (1997). Self-Efficacy: The Exercise of Control.

[12] McMullan M., Jones R., Lea S. (2012). Math anxiety, self-efficacy, and ability in British undergraduate nursing students. Res. Nurs. Health.

[13] Robb M. (2012). Self-efficacy with application to nursing education: A concept analysis. Nurs. Forum..

[14] Henderson A., Rowe J., Watson K., Hitchen-Holmes D. (2016). Graduating nurses’ self-efficacy in palliative care practice: An exploratory study. Nurse Educ. Today.

[15] Zengin N., Pınar R., Akinci A.C., Yildiz H. (2014). Psychometric properties of the self-efficacy for clinical evaluation scale in Turkish nursing students. J. Clin. Nurs..

[16] Henderson A., Harrison P., Rowe J., Edwards S., Barnes M., Henderson S. (2018). Students take the lead for learning in practice: A process for building self-efficacy into undergraduate nursing education. Nurse Educ. Pract..

[17] Oyelana O., Martin D., Scanlan J., Temple B. (2018). Learner-centred teaching in a non-learner-centred world: An interpretive phenomenological study of the lived experience of clinical nursing faculty. Nurse Educ. Today.

[18] Mackintosh-Franklin C. (2016). Pedagogical principles underpinning undergraduate nurse education in the UK: A review. Nurse Educ. Today.

[19] Betihavas V., Bridgman H., Kornhaber R., Cross M. (2016). The evidence for ‘flipping out’: A systematic review of the flipped classroom in nursing education. Nurse Educ. Today.

[20] King A., Boysen-Osborn M., Cooney R., Mitzman J., Misra A., Williams J., Dulani T., Gottlieb M. (2017). Curated collection for educators: Five key papers about the flipped classroom methodology. Cureus.

[21] Lee M.K., Chang S.J., Jang S.J. (2017). Effects of the flipped classroom approach on the psychiatric nursing practicum course. J. Korean Acad. Psych. Ment. Health Nurs..

[22] Green R.D., Schlairet M.C. (2017). Moving toward heutagogical learning: Illuminating undergraduate nursing students’ experiences in a flipped classroom. Nurse Educ. Today.

[23] Hew K.F., Lo C.K. (2018). Flipped classroom improves student learning in health professions education: A meta-analysis. BMC Med. Educ..

[24] Kim H., Jang Y. (2017). Flipped learning with simulation in undergraduate nursing education. J. Nurs. Educ..

[25] Missildine K., Fountain R., Summers L., Gosselin K. (2013). Flipping the classroom to improve student performance and satisfaction. J. Nurs. Educ..

[26] Ward M., Knowlton M.C., Laney C.W. (2018). The flip side of traditional nursing education: A literature review. Nurse Educ. Pract..

[27] Abeysekera L., Dawson P. (2015). Motivation and cognitive load in the flipped classroom: Definition, rationale and a call for research. High Educ. Res. Dev..

[28] Hu R., Gao H., Ye Y., Ni Z., Jiang N., Jiang X. (2018). Effectiveness of flipped classrooms in Chinese baccalaureate nursing education: A meta-analysis of randomized controlled trials. Int. J. Nurs. Stud..

[29] Xu P., Chen Y., Nie W., Wang Y., Song T., Li H., Li J., Yi J., Zhao L. (2019). The effectiveness of a flipped classroom on the development of Chinese nursing students’ skill competence: A systematic review and meta-analysis. Nurse Educ. Today.

[30] Chu T.L., Wang J., Monrouxe L., Sung Y.C., Kuo C.L., Ho L.H., Lin Y.E. (2019). The effects of the flipped classroom in teaching evidence based nursing: A quasi-experimental study. PLoS ONE.

[31] Zhu L., Lian Z., Engström M. (2020). Use of a flipped classroom in ophthalmology courses for nursing, dental and medical students: A quasi-experimental study using a mixed-methods approach. Nurse Educ. Today.

[32] Holland A., Smith F., McCrossan G., Adamson E., Watt S., Penny K. (2013). Online video in clinical skills education of oral medication administration for undergraduate student nurses: A mixed methods, prospective cohort study. Nurse Educ. Today.

[33] Wei J., Salvendy G. (2006). Development of a human information processing model for cognitive task analysis and design. Theor. Iss. Ergon. Sci..

[34] Jokinen P., Mikkonen I. (2013). Teachers’ experiences of teaching in a blended learning environment. Nurse Educ. Pract..

[35] Simpson V., Richards E. (2015). Flipping the classroom to teach population health: Increasing the relevance. Nurse Educ. Pract..

[36] Chan J.C.Y. (2015). Using medical incidents to teach: Effects of vicarious experience on nursing students’ self-efficacy in performing urinary catheterization. J. Nurs. Educ..

[37] Critz C.M., Knight D. (2013). Using the flipped classroom in graduate nursing education. Nurs. Educ..

[38] Maxwell K.L., Wright V.H. (2016). Evaluating the effectiveness of two teaching strategies to improve nursing students’ knowledge, skills, and attitudes about quality improvement and patient safety. Nurs. Educ. Perspect..

[39] Peisachovich E.H., Murtha S., Phillips A., Messinger G. (2016). Flipping the Classroom: A Pedagogical Approach to Applying Clinical Judgment by Engaging, Interacting, and Collaborating with Nursing Students. Learn. Teach..

